# Feasibility of online adaptive SBRT boost with 1.5-T MR-Linac for cervical cancer patients unsuitable for brachytherapy: a planning study

**DOI:** 10.1093/jrr/rrag013

**Published:** 2026-04-01

**Authors:** Kota Abe, Kei Ito, Miho Watanabe, Masato Tsuneda, Yukio Fujita, Takashi Uno

**Affiliations:** Department of Radiation Oncology, MR Linac ART Division, Graduate School of Medicine, Chiba University, 1-8-1 Inohana, Chuo-ku 260-8670, Japan; Division of Radiation Oncology, Department of Radiology, Tokyo Metropolitan Cancer and Infectious Diseases Center, Komagome Hospital, 3-18-22 Honkomagome, Bunkyo-ku 113-8677, Japan; Division of Radiation Oncology, Department of Radiology, Tokyo Metropolitan Cancer and Infectious Diseases Center, Komagome Hospital, 3-18-22 Honkomagome, Bunkyo-ku 113-8677, Japan; Diagnostic Radiology and Radiation Oncology, Graduate School of Medicine, Chiba University, 1-8-1 Inohana, Chuo-ku 260-8670, Japan; Department of Radiation Oncology, MR Linac ART Division, Graduate School of Medicine, Chiba University, 1-8-1 Inohana, Chuo-ku 260-8670, Japan; Department of Radiation Oncology, MR Linac ART Division, Graduate School of Medicine, Chiba University, 1-8-1 Inohana, Chuo-ku 260-8670, Japan; Department of Radiological Sciences, Komazawa University, 1-23-1 Komazawa, Setagaya-ku 154-8525, Japan; Diagnostic Radiology and Radiation Oncology, Graduate School of Medicine, Chiba University, 1-8-1 Inohana, Chuo-ku 260-8670, Japan

**Keywords:** MR-linac, online adaptive radiotherapy, brachytherapy ineligibility, cervical cancer, stereotactic body radiotherapy boost

## Abstract

This study aimed to evaluate the effectiveness of online adaptive treatment (ADT) planning for stereotactic body radiotherapy (SBRT) boost on a 1.5-T MR-Linac in cervical cancer patients ineligible for brachytherapy, compared with the conventional position-alignment (PA) assumed dose distributions. This retrospective analysis included 15 treatment plans from five patients, each receiving 21.0 Gy in three fractions. Daily magnetic resonance imaging (MRIs) were used to compare target and organ-at-risk (OAR) doses between ADT and PA dose distributions. The planning target volume (PTV) was defined as the high-risk clinical target volume (HR-CTV) plus a 3 mm margin. OARs included the bladder, bowel bag, and planning organ-at-risk volume (PRV) for rectum and sigmoid. Reference plans prioritized OAR constraints while maximizing target coverage; the intended PTV *D*_90%_ range was 18.9–21.6 Gy. PA doses were reconstructed by dose warping based on alignment of the anterior rectal wall between daily and pretreatment MRIs, whereas ADT plans were reoptimized on the daily MRIs. HR-CTV decreased by 1.6%–41.5% between the planning MRI and the first treatment fraction. ADT plans improved PTV *D*_90%_, achieved better target coverage and met all OAR constraints, whereas PA plans showed dose constraint exceedance across the three fractions, with mean excess doses of 6.89 ± 4.39 Gy to PRV-sigmoid and 5.76 ± 3.33 Gy to PRV-rectum. In conclusion, 1.5-T MR-Linac based SBRT boost with daily online adaptation enhanced tumor coverage and reduced OAR doses, supporting MR-guided online adaptive radiotherapy as a promising option for patients ineligible for brachytherapy.

## INTRODUCTION

The combination of pelvic external beam radiotherapy (EBRT) and brachytherapy is an established treatment for localized cervical cancer [1]. Research from the United States Surveillance, Epidemiology, and End Results Program indicates that brachytherapy is associated with improved overall survival compared to EBRT boost [2]. Consequently, brachytherapy is critical for enhancing outcomes in patients with cervical cancer. However, some patients may be ineligible for brachytherapy due to factors such as advanced age, poor general condition, or refusal to undergo the procedure [3].

Stereotactic body radiotherapy (SBRT) delivers ablative doses of radiation to tumors via extreme hypofractionation, making it a potential alternative for brachytherapy-ineligible patients [4, 5]. Several prospective and retrospective studies have reported favorable outcomes with SBRT boost [6–8], with global experts recommending a primary SBRT boost when brachytherapy is contraindicated [4, 5, 9, 10]. However, implementing a SBRT boost with conventional linear accelerators (linacs) is highly challenging due to inter- and intrafractional organ motion, as treatment is delivered by aligning SBRT plans based on simulation computed tomography (CT) using daily cone-beam CT imaging guidance. While conventional linacs can accommodate simple positional changes, they often cannot adapt to morphological changes in the tumor and surrounding organs. In particular, as cervical cancer cases are prone to morphological tumor changes, interfractional organ motion frequently cause issues between treatment sessions. Furthermore, meticulous management of organ volumes, such as bladder volume and rectal gas, is necessary to minimize intrafractional motion during treatment.

The integration of magnetic resonance imaging (MRI) and linac into MR-Linac systems has recently been adopted in clinical practice. MR-guided online adaptive radiotherapy (MRgOART) can be performed on the day of treatment to address tumor shrinkage, organ deformation, and positional changes. Furthermore, the MR-Linac allows the capture of cine MR images during irradiation, enabling real-time monitoring of organ motion during radiation delivery. Leveraging these advantages, MRgOART offers the potential to overcome the issues associated with SBRT boost for cervical cancer using conventional linacs. Building on the promising early clinical outcomes reported by Hadi et al. [11], further studies are warranted to clearly quantify the benefits of MRgOART compared with conventional SBRT-boost. Therefore, in this study, we conducted a planning study analysis on patients with cervical cancer treated with SBRT boost to compare adaptive MR-Linac treatment plans with conventional linac plans and evaluate their effectiveness.

## MATERIALS AND METHODS

### Patients

This study was approved by the Institutional Ethics Committee of our hospital (approval number M10434). Written informed consent was obtained from all patients. In this study, five patients were enrolled for the analysis of their 15 treatment plans. Patient characteristics are shown in Table 1. We retrospectively evaluated patients with cervical cancer who were ineligible for brachytherapy and underwent SBRT boost using MRgOART.

**Table 1:** Patient characteristics

Patient	Age [years]	Clinical Stage	Histological type	Whole pelvic irradiation	Tumor volume before SBRT [cc]
1	33	T4aN1M0	Squamous cell carcinoma	CCRT	171.6
2	66	T3bN0M0	Squamous cell carcinoma	RT alone	115.7
3	90	T3bN1M0	Squamous cell carcinoma	RT alone	71.8
4	42	T4aN0M0	Adenocarcinoma	CCRT	90.8
5	81	T2aN1M0	Carcinoma admixed with neuroendocrine carcinoma	RT alone	37.0

Abbreviations: CCRT, concurrent chemoradiotherapy; RT, radiotherapy; SBRT, stereotactic body radiotherapy

### SBRT plan protocol

This planning study focused only on SBRT boost treatment plans. The high-risk clinical target volume (HR-CTV) included the whole cervix, gross tumor, and suspected residual tumor at the time of SBRT. The contour was set as the planning target volume (PTV) with HR-CTV + 3 mm. The bladder, bowel bag, and planning organ at risk volume (PRV) were created with a 3 mm margin added to the rectum (PRV rectum) and sigmoid colon (PRV sigmoid). The PTV for dose evaluation (PTVeval) was defined as subtracting the organ-at-risk (OAR) contours themselves from the PTV. The SBRT boost of 21 Gy in three fractions was planned with MR-linac Elekta Unity (Elekta AB, Stockholm, Sweden) using step-and-shoot intensity modulated radiotherapy (IMRT) with the Monaco treatment planning system (Elekta AB, Stockholm, Sweden). The optimal dose to 90% (*D*_90%_) of the PTVeval was required to be between 90% and 103% of the prescribed dose, and the maximum dose (*D*_0.03 cc_) was required to be between 140% and 160% of the prescribed dose. The mandatory dose constraint for PTVeval *D*_90%_ was set to a minimum of 70% of the prescribed dose. The optimization was performed with the highest priority given to OAR dose constraints while attempting to maximize target dose delivery under these conditions. The per-fraction dose constraints for OARs were defined based on a total prescription dose of 21 Gy in three fractions: 7 Gy for the bladder, 5.0 Gy for bowel bag, and 5.5 Gy for PRV rectum and PRV sigmoid. When creating the reference SBRT boost plan, the maximum number of segments was set to 70–100, and 11–13 beams were used. The beam arrangement excluded the couch edge and the MRI cryostat pipes. A dose grid of 2.5 mm was used for all treatment plans. In the adaptive plan, the maximum number of segments, beams, and beam angles were kept consistent with those of the reference plan.

### Adaptive treatment plan and position-alignment assumed dose distributions creation

**Fig. 1:**
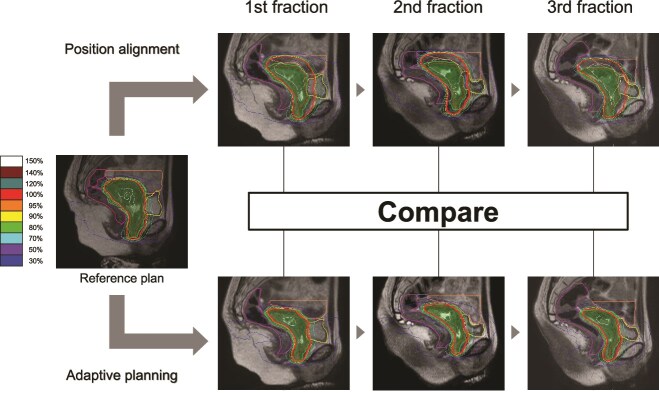
Workflow for comparing position alignment and adaptive treatment plans. The planning target volume in each treatment plan is represented by the green region.

The planning MRI for the reference SBRT boost plan was performed 2 weeks before the first SBRT session. Figure 1 shows the workflow of this study. Position-alignment (PA) assumed dose distributions were used to simulate the actual position alignment during treatment with conventional linacs. First, the anterior rectal walls on the pretreatment MRI and the three daily MRIs were aligned through a rigid registration process. Finally, the PA plan doses were reproduced on the daily MRI scans by applying dose warping based on the registration information.

Adaptive treatment (ADT) plans were created for each of the three MR images obtained during treatment sessions, following a routine clinical workflow. The treatment plan was adjusted by modifying the IMRT constraints if necessary. To evaluate the dosimetric effect, target and OARs doses were compared between the PA and ADT plans.

## RESULTS

**Fig 2:**
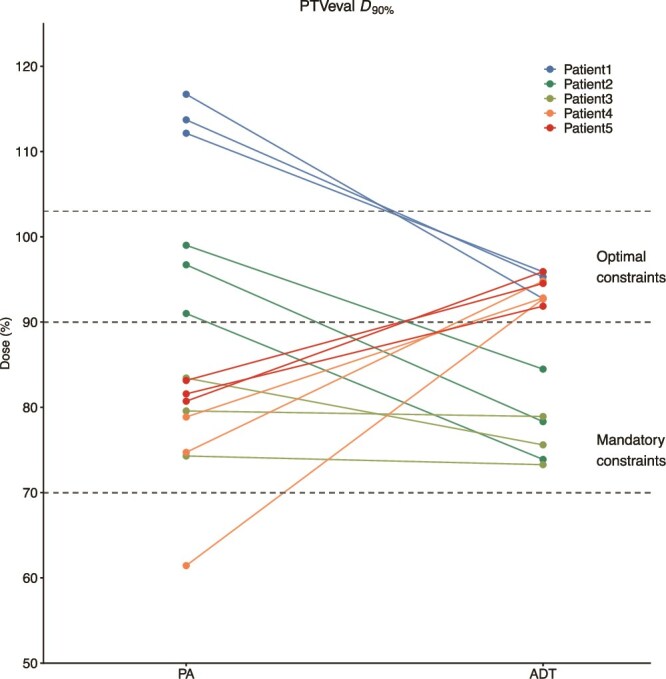
Comparison of PTV for dose evaluation (PTVeval) D90% between position alignment (PA) virtual plans and adaptive treatment planning (ADT) methods. Each line represents the dose transition from PA to ADT plans for each fraction across five patients. The horizontal dashed lines indicate the optimal (90%–103% of the prescribed dose) and mandatory (70%–90% of the prescribed dose) dose constraints.

**Fig. 3:**
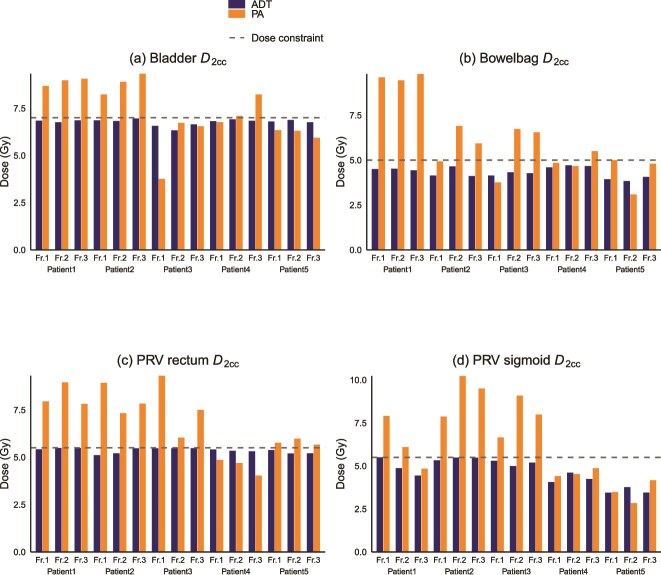
Comparison of *D*_2 cc_ values for organs at risk (OARs) between the position alignment (PA) virtual plan and adaptive treatment planning (ADT). Each bar represents the *D*_2 cc_ for each fraction across five patients. (a) Bladder, (b) Bowel bag, (c) Planning organ-at-risk volume (PRV) rectum, and (d) PRV sigmoid. Orange bars indicate PA plans, and blue bars indicate ADT plans. The horizontal dashed lines represent the per-fraction dose constraints for each organ (7 Gy for the bladder, 5 Gy for the bowel bag, and 6 Gy for the PRV rectum and PRV sigmoid).

The reduction in HR-CTV between the planning MRI and the first fraction varied among the analyzed patients, with rates of 35.4%, 41.5%, 17.5%, 4.0%, and 1.6% in patients 1 to 5, respectively. Figure 2 shows that ADT plans improved PTVeval *D*_90%_ compared with PA plans. All cases satisfied the mandatory constraint (70%–90% of the prescribed dose), with a marked increase in cases achieving optimal coverage. Figure 3 compares the *D*_₂ cc_ values of OARs between PA and ADT methods across five patients and three treatment fractions. ADT plans consistently showed lower doses compared with PA plans, with improved compliance to dose constraints for the bladder, bowel bag, PRV rectum, and PRV sigmoid. When evaluating the cumulative dose over three fractions, PA plans exceeded OAR dose constraints for the bladder, bowel bag, PRV rectum, and PRV sigmoid in three, four, four, and three patients, respectively, whereas no violations occurred in the ADT plans. Among patients who exceeded dose constraints in the PA plan, the mean excess doses were 4.10 ± 2.60 Gy for the bladder, 4.67 ± 6.24 Gy for the bowel bag, 5.76 ± 3.33 Gy for the PRV rectum, and 6.89 ± 4.39 Gy for the PRV sigmoid.

## DISCUSSION

This study compared target and OARs doses between PA and ADT plans using MR-Linac. The findings showed that ADT plans improved target dose coverage and reduced the doses to OARs compared with conventional PA-based treatment plans in SBRT boost for cervical cancer, regardless of tumor shrinkage.

In patients with substantial tumor shrinkage (i.e. ≥15% HR-CTV reduction; patients 1–3), the PA plan often exceeded PTV and OAR dose constraints. In contrast, the ADT plan achieved PTV coverage within the optimal or mandatory ranges while maintaining OAR doses within their respective constraints. Notably, PTVeval D_90%_ improved with the ADT plan compared with the PA plan in patients with substantial tumor shrinkage and in those with minimal tumor shrinkage (i.e. <5% HR-CTV reduction; patients 4 and 5). This suggests that the effectiveness of the ADT approach is not solely dependent on tumor volume reduction, but rather reflects its capacity to adapt to treatment-day anatomical changes, such as altered spatial relationships between the tumor and OARs.

Although this study quantitatively evaluated the benefits of adaptive treatment, MR-Linac offers additional advantages for cervical cancer in the following respects: first, MRI is highly advantageous in managing cervical cancer because it allows for precise assessment of tumor response and plays a crucial role in defining the HR-CTV in brachytherapy [12]. Second, MR-Linac enables real-time tumor monitoring during irradiation using cineMRI. This capability allows for precise beam control, even in cases of internal organ movement during irradiation (e.g. rectal gas occurrence) [13]. These advantages suggested that SBRT boost with MR-Linac could be a viable alternative for patients with cervical cancer for whom brachytherapy is not feasible.

A limitation of this study is that the findings are based on only five cases. The small sample size indicates that the results should be interpreted with caution, and further research with larger patient cohorts is needed to confirm these findings. To clarify whether the dosimetric advantages translate into clinical outcomes, we have initiated a clinical trial (trial registry number UMIN000055987). In conclusion, our planning study indicated that SBRT-boost with an MR-Linac offered a substantial improvement over conventional position alignment-based techniques, showing the capacity to simultaneously improve tumor coverage and reduce doses to OARs. The superior soft-tissue visualization of the MR-Linac provided a distinct advantage, particularly in challenging cases such as cervical cancer, where tumor shrinkage during the treatment preparation period and organ motion during irradiation have historically rendered brachytherapy the only feasible option. By enabling precise daily adaptations, MRgOART had the potential to provide a safer and more effective method for delivering SBRT-boost. These findings suggested that MRgOART could be considered a promising alternative for patients who are ineligible for brachytherapy, potentially expanding their treatment opportunities.

## Data Availability

Research data are not available at this time.
